# Dichotomous versus 5-scale grading system for the interpretation of the point-of-care immunoassay for tear matrix metalloproteinase-9 in dry eye

**DOI:** 10.1038/s41598-023-32928-3

**Published:** 2023-04-13

**Authors:** Ja Young Oh, Yeoun Sook Chun, Kyoung Woo Kim

**Affiliations:** Department of Ophthalmology, Chung-Ang University College of Medicine, Chung-Ang University Hospital, 102 Heukseok-ro, Dongjak-gu, Seoul, 06973 Republic of Korea

**Keywords:** Biomarkers, Diseases, Medical research

## Abstract

In this study, we compared the dichotomous and 5-scale grading systems for point-of-care immunoassay of tear matrix metalloproteinase (MMP)-9 in dry eye disease (DED) patients and identified the optimal dichotomous system to correlate with DED parameters. We included 167 DED patients without primary Sjogren's syndrome (pSS) (Non-SS DED) and 70 DED patients with pSS (SS DED). We graded MMP-9 expression in InflammaDry® (Quidel, San Diego, CA, USA) using a 5-scale grading system and dichotomous grading systems with four different cut-off grades (D1 to D4 systems). The only DED parameter that showed a significant correlation with the 5-scale grading method was tear osmolarity (Tosm). In both groups, subjects with positive MMP-9 had lower tear secretion and higher Tosm than those with negative MMP-9 according to the D2 dichotomous system. Tosm determined D2 positivity at cutoffs > 340.5 and > 317.5 mOsm/L in the Non-SS DED and SS DED groups, respectively. Tear secretion < 10.5 mm or tear break-up time < 5.5 s stratified D2 positivity in the Non-SS DED group. In conclusion, the dichotomous grading system of InflammaDry reflects ocular surface indices better than the 5-scale grading system and may be more practical in real clinical circumstances.

## Introduction

Matrix metalloproteinases (MMPs) are proteolytic enzymes that play an important role in inflammation-induced wound healing. MMP-9, in particular, has been known to degrade tight junctions, leading to disruption of barrier function in the corneal epithelium^1^. As such, MMP-9 may contribute to the pathogenesis of various conditions, including dry eye disease (DED), blepharitis, sterile corneal ulceration, ocular allergy, fungal keratitis, burns, advanced keratoconus with an irregular surface, active pterygia, and conjunctivochalasis^2–9^.

Fortunately, a commercial point-of-care immunoassay for MMP-9 measurement in tears is now available, known as InflammaDry® (Quidel, San Diego, CA, USA), which was introduced in 2013^10^. The InflammaDry test is a disposable and semi-quantitative test that displays the level of MMP-9 through a red-colored band within 10 min of its application to the inferior conjunctival fornix tear film, and is widely used for evaluating dry eye disease (DED). It has demonstrated high positive and negative agreement for confirming suspected DED^11^, and was found to be useful in verifying the effectiveness of topical anti-inflammatory cyclosporin A^12^. Additionally, the InflammaDry test results correlated well with those of other dry eye tests in confirmed dry eye patients^13^.

Although it was recently discovered that the 5-scale grading system is sensitive to changes in MMP-9 concentrations in tears and has favorable reliability and accuracy^14^, such semi-quantitative grading of results remains controversial. Additionally, many clinicians still intuitively judge the results using a dichotomous approach, distinguishing between negative and positive results^15,16^. Nevertheless, determining the optimal single thickness of a red line to distinguish positive from negative results and correlate well with various pre-established dry eye parameters remains a challenge.

To address this issue, we conducted a comparative analysis of dichotomous interpretation with varying cut-off thicknesses of readout lines and a 5-scale grading system in InflammaDry tests. Our goal was to identify the optimal system that would enable us to effectively correlate the test results with objective dry eye parameters in patients with DED.

## Results

### Demographics, MMP-9, and clinical parameters of DED

Table 1 presents the demographics, MMP-9 levels, and clinical parameters of DED in each group. The study included a total of 464 eyes from 237 patients with DED, of which 167 had DED without primary Sjogren’s syndrome (pSS) (i.e. non-SS DED group), and 70 had DED with pSS (i.e. SS DED group). The mean age in the non-SS DED and SS DED groups was 59.7 ± 15.0 years and 54.1 ± 12.7 years, respectively (*P* = 0.002). The female gender was more prevalent in the SS DED group (92.9%) than in the non-SS DED group (69.5%, *P* < 0.001). Among the DED parameters, corneal erosion score, Sjogren’s International Collaborative Clinical Alliance (SICCA) ocular staining score (OSS), Schirmer I, and tear break-up time (BUT) were significantly worse in the SS DED group (*P* < 0.001, *P* < 0.001, *P* < 0.001, and *P* = 0.003, respectively). However, the ocular surface disease index (OSDI) score was higher in the non-SS DED group (*P* = 0.001). Corneal sensitivity, meibomian gland dysfunction (MGD) grades, and tear osmolarity (Tosm) did not differ significantly between the two groups. In terms of the InflammaDry test, the grades according to the 5-scale grading system were significantly higher in the SS DED group (*P* < 0.001). Moreover, positive patients by D2, D3, and D4 dichotomous systems were significantly higher in the SS-DED group (*P* < 0.001, *P* < 0.001, and *P* = 0.030, respectively).

**Table 1:** Demographics and ocular surface indices in subjects with dry eye disease (DED) with and without primary Sjogren’s syndrome (pSS).

Variables	Group		*P* value
	Non-SS DED	SS DED	
Total No. of patients (eyes)	167 (325)	70 (139)	–
Demographics			
Age	59.7 $\pm$ 15.0	54.1 $\pm$ 12.7	0.002*
Male/female (% female)	51/116 (69.5%)	5/65 (92.9%)	$<$ 0.001*
Ocular surface indices			
Corneal erosion (NEI score)	1.1 $\pm$ 1.7	2.2 $\pm$ 2.1	$<$ 0.001*
SICCA OSS	0.8 $\pm$ 1.4	2.8 $\pm$ 2.4	$<$ 0.001*
Schirmer I without anesthesia (mm)	11.7 $\pm$ 8.5	7.8 $\pm$ 7.0	$<$ 0.001*
Tear BUT (s)	6.0 $\pm$ 2.1	5.4 $\pm$ 2.5	0.003*
Corneal sensitivity (cm)	5.9 $\pm$ 0.3	5.8 $\pm$ 0.8	0.105
MG expressibility (Gr)	1.4 $\pm$ 0.6	1.4 $\pm$ 0.6	0.982
Meibum quality (Gr)	1.5 $\pm$ 0.7	1.4 $\pm$ 0.8	0.161
OSDI (score)	43.4 $\pm$ 18.9	36.6 $\pm$ 22.1	0.001*
Tear osmolarity (mOsm/L)	316.1 $\pm$ 21.2	316.0 $\pm$ 19.4	0.958
Tear MMP-9			
5-scale grading (Gr) system	1.5 $\pm$ 1.1	2.1 $\pm$ 1.2	< 0.001*
Dichotomous system			
D1			
N	50 (15.4%)	18 (12.9%)	0.497
P	275 (84.6%)	121 (87.1%)	
Total	325	139	
D2			
N	178 (54.8%)	45 (32.4%)	< 0.001*
P	147 (45.2%)	94 (67.6%)	
Total	325	139	
D3			
N	267 (82.2%)	86 (61.9%)	< 0.001*
P	58 (17.8%)	53 (38.1%)	
Total	325	139	
D4			
N	308 (94.8%)	124 (89.2%)	0.030*
P	17 (5.2%)	15 (10.8%)	
Total	325	139	

*P $<$ 0.05. Gr, grade. OSDI, ocular surface disease index. OSS, ocular staining score. BUT, break-up time. MG, meibomian gland. MMP-9, matrix metalloproteinase 9. N, negative. P. positive.

Significant are in value [bold].

### Difference of ocular surface indices according to the expression of MMP-9 in tear

We evaluated ocular surface indices for DED depending on the positivity of tear MMP-9 expression by the D1 to D4 dichotomous system in both groups, as shown in Tables 2, 3, 4 and 5. All ocular surface indices were not different between positive and negative MMP-9 in tears by D1 and D4 systems in both groups (Tables 2 and 5). However, according to the D2 system, subjects with positive tear MMP-9 had lower tear secretion and higher Tosm in both groups and had a shorter BUT in the Non-SS DED group compared to subjects with negative tear MMP-9 (Table 3). According to the D3 system, subjects with positive tear MMP-9 in the SS DED group had higher tear osmolarity than those with negative tear MMP-9 (*P* = 0.020, Table 4).

**Table 2:** Ocular surface indices according to D1 dichotomous system for tear matrix metalloproteinase 9 (MMP-9) expression in subjects with dry eye disease (DED) with and without primary Sjogren’s syndrome (pSS).

Variables	Group					
	Non-SS DED			SS DED		
	Tear MMP-9 by D1 dichotomous system		*P* value	Tear MMP-9 by D1 dichotomous system		*P* value
	N	P		N	P	
No. of patients (eyes)	27 (50)	140 (275)	–	7 (18)	63 (121)	–
Ocular surface indices						
Corneal erosion (NEI score)	1.1 $\pm$ 1.8	1.1 $\pm$ 1.6	0.967	1.3 $\pm$ 1.7	2.3 $\pm$ 2.1	0.073
SICCA OSS	0.7 $\pm$ 1.1	0.8 $\pm$ 1.4	0.538	2.5 $\pm$ 2.7	2.8 $\pm$ 2.3	0.572
Schirmer I without anesthesia (mm)	10.3 $\pm$ 6.8	11.9 $\pm$ 8.6	0.411	6.5 $\pm$ 5.9	8.0 $\pm$ 7.2	0.145
Tear BUT (s)	6.1 $\pm$ 2.0	6.0 $\pm$ 2.1	0.716	6.3 $\pm$ 2.5	5.2 $\pm$ 2.5	0.178
Corneal sensitivity (cm)	5.9 $\pm$ 0.2	5.9 $\pm$ 0.3	0.660	5.2 $\pm$ 2.0	5.9 $\pm$ 0.3	0.275
MG expressibility (Gr)	1.3 $\pm$ 0.5	1.4 $\pm$ 0.6	0.098	1.4 $\pm$ 0.5	1.4 $\pm$ 0.6	0.913
Meibum quality (Gr)	1.5 $\pm$ 0.5	1.5 $\pm$ 0.7	0.664	1.2 $\pm$ 0.7	1.5 $\pm$ 0.8	0.136
OSDI (score)	41.2 $\pm$ 18.0	43.8 $\pm$ 19.1	0.458	38.4 $\pm$ 25.3	36.3 $\pm$ 21.7	0.771
Tear osmolarity (mOsm/L)	314.7 $\pm$ 19.4	316.5 $\pm$ 21.7	0.617	315.5 $\pm$ 26.6	316.1 $\pm$ 17.6	0.937

N, negative. P, positive. Gr, grade. OSDI, ocular surface disease index. OSS, ocular staining score. BUT, break-up time. MG, meibomian gland.

**Table 3:** Ocular surface indices according to D2 dichotomous system for tear matrix metalloproteinase 9 (MMP-9) expression in subjects with dry eye disease (DED) with and without primary Sjogren’s syndrome (pSS).

Variables	Group					
	Non-SS DED			SS DED		
	Tear MMP-9 by D2 dichotomous system		*P* value	Tear MMP-9 by D2 dichotomous system		*P* value
	N	P		N	P	
No. of patients (eyes)	90 (178)	77 (147)		20 (45)	50 (94)	–
Ocular surface indices						
Corneal erosion (NEI score)	1.0 $\pm$ 1.6	1.1 $\pm$ 1.8	0.848	1.8 $\pm$ 1.9	2.3 $\pm$ 2.2	0.325
SICCA OSS	0.7 $\pm$ 1.3	0.9 $\pm$ 1.4	0.592	3.0 $\pm$ 2.6	2.7 $\pm$ 2.2	0.721
Schirmer I without anesthesia (mm)	12.6 $\pm$ 8.9	10.5 $\pm$ 7.5	0.027*	10.3 $\pm$ 9.6	6.6 $\pm$ 5.0	0.003*
Tear BUT (s)	6.3 $\pm$ 2.0	5.7 $\pm$ 2.1	0.012*	6.0 $\pm$ 2.7	5.0 $\pm$ 2.3	0.126
Corneal sensitivity (cm)	5.9 $\pm$ 0.2	5.9 $\pm$ 0.3	0.727	5.9 $\pm$ 0.3	5.8 $\pm$ 0.9	0.988
MG expressibility (Gr)	1.4 $\pm$ 0.5	1.5 $\pm$ 0.6	0.168	1.4 $\pm$ 0.5	1.4 $\pm$ 0.6	0.691
Meibum quality (Gr)	1.5 $\pm$ 0.6	1.5 $\pm$ 0.7	0.849	1.3 $\pm$ 0.8	1.5 $\pm$ 0.8	0.364
OSDI (score)	43.1 $\pm$ 18.8	43.8 $\pm$ 19.2	0.857	35.2 $\pm$ 20.0	37.2 $\pm$ 23.2	0.886
Tear osmolarity (mOsm/L)	313.1 $\pm$ 19.7	320.8 $\pm$ 22.7	0.008*	310.2 $\pm$ 23.7	320.0 $\pm$ 14.8	0.047*

*P $<$ 0.05. N, negative. P, positive. Gr, grade. OSDI, ocular surface disease index. OSS, ocular staining score. BUT, break-up time. MG, meibomian gland.

Significant are in value [bold].

**Table 4:** Ocular surface indices according to D3 dichotomous system for tear matrix metalloproteinase 9 (MMP-9) expression in subjects with dry eye disease (DED) with and without primary Sjogren’s syndrome (pSS).

Variables	Group					
	Non-SS DED			SS DED		
	Tear MMP-9 by D3 dichotomous system		*P* value	Tear MMP-9 by D3 dichotomous system		*P* value
	N	P		N	P	
No. of patients (eyes)	136 (267)	31 (58)		43 (86)	27 (53)	–
Ocular surface indices						
Corneal erosion (NEI score)	1.1 $\pm$ 1.6	1.0 $\pm$ 1.9	0.835	2.3 $\pm$ 2.3	1.9 $\pm$ 1.8	0.431
SICCA OSS	0.8 $\pm$ 1.3	0.9 $\pm$ 1.6	0.667	3.1 $\pm$ 2.5	2.2 $\pm$ 2.0	0.060
Schirmer I without anesthesia (mm)	12.1 $\pm$ 8.6	9.8 $\pm$ 7.0	0.108	8.4 $\pm$ 7.7	6.7 $\pm$ 5.7	0.162
Tear BUT (s)	6.0 $\pm$ 2.1	5.8 $\pm$ 2.1	0.573	5.7 $\pm$ 2.6	4.8 $\pm$ 2.3	0.142
Corneal sensitivity (cm)	5.9 $\pm$ 0.3	6.0 $\pm$ 0.1	0.491	5.9 $\pm$ 0.3	5.7 $\pm$ 1.1	0.500
MG expressibility (Gr)	1.4 $\pm$ 0.6	1.5 $\pm$ 0.6	0.324	1.4 $\pm$ 0.6	1.4 $\pm$ 0.7	0.885
Meibum quality (Gr)	1.5 $\pm$ 0.6	1.5 $\pm$ 0.8	0.555	1.4 $\pm$ 0.8	1.5 $\pm$ 0.8	0.675
OSDI (score)	42.7 $\pm$ 17.9	46.9 $\pm$ 23.5	0.197	36.5 $\pm$ 21.1	36.7 $\pm$ 24.1	0.697
Tear osmolarity (mOsm/L)	315.1 $\pm$ 20.6	322.9 $\pm$ 24.2	0.062	312.4 $\pm$ 20.6	325.8 $\pm$ 11.2	0.020*

*P $<$ 0.05. N, negative. P, positive. Gr, grade. OSDI, ocular surface disease index. OSS, ocular staining score. BUT, break-up time. MG, meibomian gland.

Significant are in value [bold].

**Table 5:** Ocular surface indices according to D4 dichotomous system for tear matrix metalloproteinase 9 (MMP-9) expression in subjects with dry eye disease (DED) with and without primary Sjogren’s syndrome (pSS).

Variables	Group					
	Non-SS DED			SS DED		
	Tear MMP-9 by D4 dichotomous system		*P* value	Tear MMP-9 by D4 dichotomous system		*P* value
	N	P		N	P	
No. of patients (eyes)	157 (308)	10 (17)	–	62 (124)	8 (15)	–
Ocular surface indices						
Corneal erosion (NEI score)	1.1 $\pm$ 1.7	0.3 $\pm$ 0.7	0.076	2.1 $\pm$ 2.1	2.5 $\pm$ 2.3	0.547
SICCA OSS	0.8 $\pm$ 1.4	0.3 $\pm$ 0.9	0.193	2.8 $\pm$ 2.4	2.5 $\pm$ 2.1	0.732
Schirmer I without anesthesia (mm)	11.6 $\pm$ 8.3	13.3 $\pm$ 10.2	0.495	8.0 $\pm$ 7.3	6.0 $\pm$ 4.6	0.256
Tear BUT (s)	6.0 $\pm$ 2.1	6.2 $\pm$ 1.3	0.681	5.4 $\pm$ 2.4	4.9 $\pm$ 3.3	0.165
Corneal sensitivity (cm)	5.9 $\pm$ 0.3	5.9 $\pm$ 0.2	0.232	5.8 $\pm$ 0.8	5.7 $\pm$ 0.4	0.167
MG expressibility (Gr)	1.4 $\pm$ 0.6	1.5 $\pm$ 0.5	0.725	1.4 $\pm$ 0.6	1.5 $\pm$ 0.7	0.910
Meibum quality (Gr)	1.5 $\pm$ 0.7	1.6 $\pm$ 0.9	0.755	1.4 $\pm$ 0.8	1.5 $\pm$ 0.9	0.875
OSDI (score)	43.1 $\pm$ 18.7	48.9 $\pm$ 23.7	0.560	36.1 $\pm$ 21.4	40.6 $\pm$ 29.0	0.799
Tear osmolarity (mOsm/L)	316.0 $\pm$ 21.0	320.1 $\pm$ 26.0	0.547	315.3 $\pm$ 20.2	323.0 $\pm$ 4.4	0.400

N, negative. P, positive. Gr, grade. OSDI, ocular surface disease index. OSS, ocular staining score. BUT, break-up time. MG, meibomian gland.

In addition, we verified the relationship between MMP-9 expression grades in tears according to the 5-scale grading system and each ocular surface index of DED. Only Tosm showed a significant correlation with the grades of tear MMP-9 in both groups (Non-SS DED group: *r* = 0.139 and *P* = 0.041; SS DED group: *r* = 0.297 and *P* = 0.027, Table 6).

**Table 6:** Correlations of ocular surface indices with the grades of tear matrix metalloproteinase 9 (MMP-9) expression according to the 5-scale grading system in subjects with dry eye disease (DED) with and without primary Sjogren’s syndrome (pSS).

Tear MMP-9 Gr. *versus* ocular surface indices	Group			
	Non-SS DED		SS DED	
	Correlation coefficient	*P* value	Correlation coefficient	*P* value
Corneal erosion (NEI score)	− 0.012	0.846	0.044	0.612
SICCA OSS	0.024	0.698	− 0.086	0.332
Schirmer I without anesthesia (mm)	− 0.073	0.190	− 0.105	0.220
Tear BUT (s)	− 0.112	0.082	− 0.198	0.063
Corneal sensitivity (cm)	0.018	0.824	− 0.057	0.629
MG plugging (Gr)	0.098	0.084	0.019	0.828
Meibum quality (Gr)	0.005	0.935	0.086	0.319
OSDI (score)	0.037	0.550	0.011	0.916
Tear osmolarity (mOsm/L)	0.139	0.041*	0.297	0.027*

*P $<$ 0.05. Gr, grade. OSDI, ocular surface disease index. OSS, ocular staining score. BUT, break-up time. MG, meibomian gland.

Significant are in value [bold].

### ROC curve analysis for the positive tear MMP-9 by D2 system

**Figure 1:**
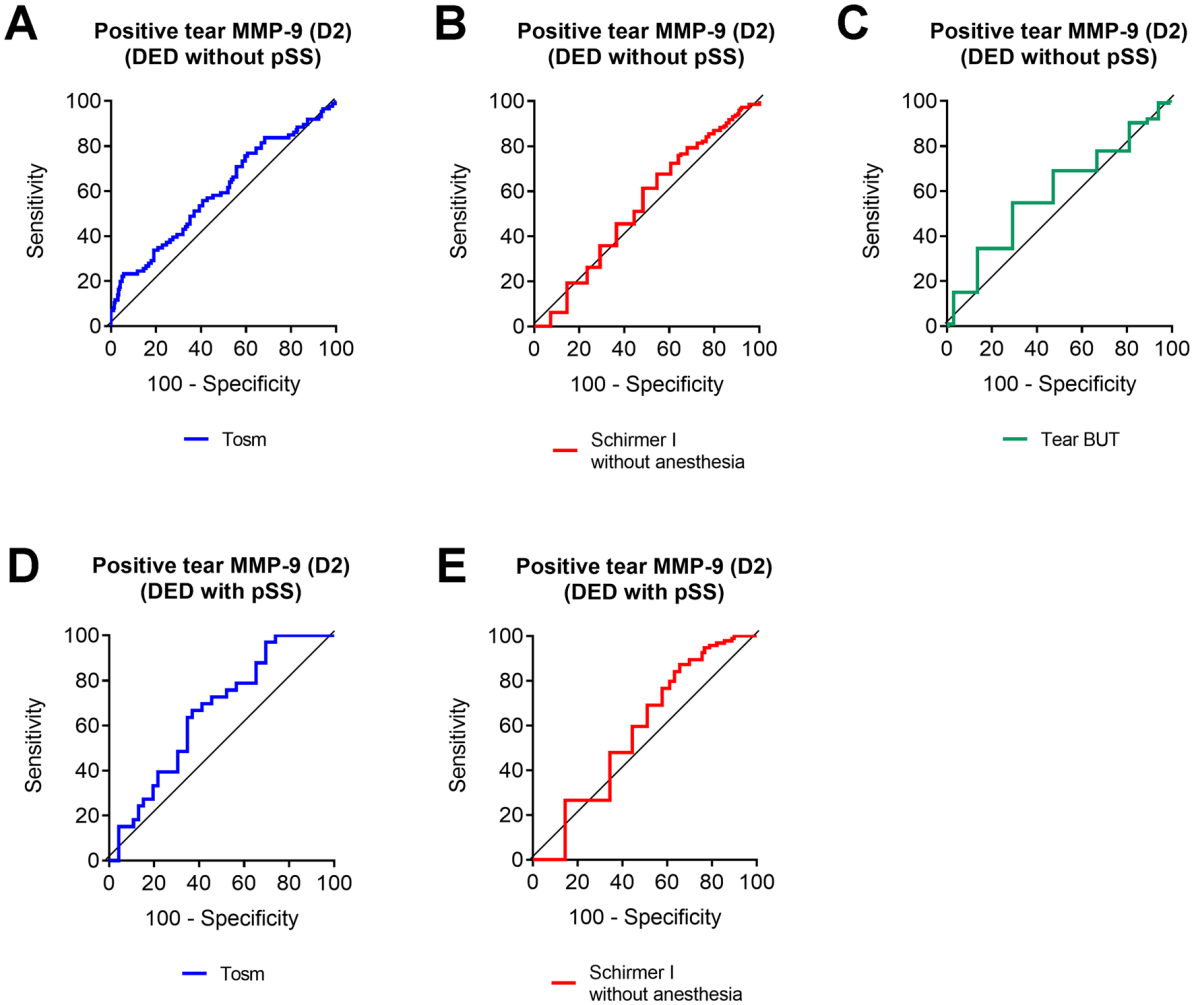
Receiver operating characteristic (ROC) curve analysis of tear osmolarity (Tosm), Schirmer I without anesthesia, and/or tear break-up time (BUT) in dry eye disease (DED) patients with and without primary Sjogren’s syndrome (pSS) and their association with positive tear matrix metalloproteinase (MMP)-9 by D2 dichotomous grading system.

Using the D2 system, we stratified the most ocular surface indices according to the positivity of tear MMP-9 (Table 3). We then performed ROC curve analysis of positive tear MMP-9 by D2 system with Tosm, tear secretion, and/or tear BUT in both groups. The ROC curves showed that Tosm best distinguished patients with positive MMP-9 by D2 system from those with negative MMP-9 in both groups (AUC = 0.599 in the Non-SS DED group and AUC = 0.657 in the SS DED group, Table 7 and Fig. 1). A Schirmer I value of 10.5 mm and a tear BUT value of 5.5 s also stratified patients by the predictability of positive tear MMP-9 by D2 system (Table 7 and Fig. 1).

**Table 7:** ROC curve analysis of positive tear matrix metalloproteinase 9 (MMP-9) according to D2 dichotomous system with the ocular surface indices in subjects with dry eye disease (DED) with and without primary Sjogren’s syndrome (pSS).

Group	D2 dichotomous system						
	Ocular surface indices	AUC	95% Confidence interval	*P* value	Cutoff	Sensitivity (%)	Specificity (%)
Non-SS DED	Tear osmolarity (mOsm/L)	0.599	0.521–0.677	0.014*	$>$ 340.5	23.3	90.1
	Schirmer I without anesthesia (mm)	0.565	0.503–0.628	0.043*	$<$ 10.5	67.6	45.5
	Tear BUT (s)	0.592	0.520–0.664	0.013*	$<$ 5.5	54.9	64.4
SS DED	Tear osmolarity (mOsm/L)	0.657	0.506–0.808	0.048*	$>$ 317.5	66.7	60.9
	Schirmer I without anesthesia (mm)	0.576	0.466–0.686	0.146	$<$ 10.5	87.2	33.3

*P $<$ 0.05. AUC, area under curve. BUT, break-up time.

Significant are in value [bold].

## Discussion

In this study, we compared the 5-scale grading system and the dichotomous grading system of the point-of-care immunoassay for tear MMP-9. Furthermore, we suggested the optimal dichotomous system and identified the relevant cut-off values of Tosm, tear wettability length by Schirmer I, and tear BUT to specify positive MMP-9 expression in tear samples. The grade 2 density by the 5-scale grading system distinguished the ocular surface indices the most. Therefore, we suggested using such a D2 dichotomous grading system as a useful method to reflect the desiccating stress and tear secretion in DED patients.

Previously, MMP-9 in tears has been suggested as a reliable indicator for inflammation in DED, using experimental methods such as MMP activity assay kits, real-time polymerase chain reaction for RNA level of expression, double sandwich enzyme-linked immunosorbent assay kits, and SDS-gelatin polyacrylamide gel electrophoresis^9,17–19^. Fortunately, InflammaDry was recently released to evaluate tear MMP-9 in clinical circumstances. It is easy to use and takes only a few minutes to get results. Like many other immunoassays, InflammaDry offers useful information on the current ocular surface inflammatory status and provides guidance on when to start anti-inflammatory treatment^13,20^. Because InflammaDry has high sensitivity and specificity and high agreement with other diagnostic tools for DED^10–13^, it is now accepted as one of the favorable test modalities for DED.

Although it is easy to identify the redline in the readout window of the kit, there is still a debate on which thickness is proper for the positive interpretation of the significant existence of MMP-9 in tear. In our recent study, the band density continued to increase up to a maximal concentration of 5000 ng/mL according to the calibration curve. The difference of grades reflected the change of MMP-9 concentrations sensitively, especially between grade 2 and 4 according to the 5-scale grading system^14^. Although the subjective 5-scale grading system in the point-of-care MMP-9 immunoassay is a reliable method with acceptable accuracy as such^14^, in this study, the gradient of 5-scale grades of tear MMP-9 did not reflect the gradients of well-established ocular surface indices in both groups, except tear osmolarity (Table 6). On the other hand, the D2 dichotomous system of tear MMP-9 stratified tear secretion as well as Tosm in both groups. Tear BUT was significantly different between negative and positive MMP-9 by D2 system in the SS DED group. This was a different result compared to the results according to D1, D3, D4 systems. Although we proposed the cut-off values of Tosm to predict positive MMP-9 by D2 system in the Non-SS DED group with high specificity, the sensitivity was very low, and the cut-off value of 340.5 mOsm/L seems too high to offer helpful clinical guidance to evaluate DED. Moreover, the significant AUC values for the stratification of ocular surface indices were quite low with unfavorable validity values. We suggest that it might be because DED is a multifactorial disease, and the alteration of various parameters may follow one after another.

Nevertheless, this study is significant as it identified the D2 system as the most reliable grading system of InflammaDry, reflecting changes in Tosm and tear secretion more accurately than diverse ocular surface indices used to evaluate the severity of DED. The D2 system may be advantageous as it only requires one standard photograph to distinguish positive results. Although it is unclear why the D2 system discriminated only a few indices, it is established that high tear osmolarity caused by reduced tear production from the lacrimal gland or excessive evaporation on the ocular surface initiates inflammatory cascades^21^.

The manufacturer defines a positive MMP-9 result as ≥ 40 ng/mL, even if the test strip's red line is faint, incomplete, or uneven. However, a positive result does not always indicate altered DED clinical parameters. In this study, we propose a modified positive cut-off value of MMP-9 for the D2 system based on tear secretion and instability. Although we do not indicate the actual cut-off value of MMP-9 concentration in the D2 system, our previous study showed that the positive cut-off range for recombinant human pro-MMP-9 concentration in the D2 system relevant to grade 2 on a 5-scale grading system was 100 to 2500 ng/mL^14^. Thus, we suggest that clinical parameter changes for DED occur at MMP-9 concentrations much higher than 40 ng/mL.

In conclusion, we compared the grading system with different cut-off values of InflammaDry and identified that the positive tear MMP-9 by the D2 dichotomous grading system of InflammaDry reflected the hyperosmolar and desiccating stress on the ocular surface. As the InflammaDry test is semi-quantitative, the dichotomous grading rather than the 5-scale grading reflects the ocular surface indices more accurately and may be more practical in real clinical circumstances.

## Methods

This was a retrospective cross-sectional study that aimed to correlate various tear MMP-9 grading systems with clinical ocular surface parameters in subjects with DED. The study was approved by the Chung-Ang University Hospital Institutional Review Board (IRB) and the informed consent was waived by an IRB (Approval No.: 2004-003-19308). It properly followed the tenets of the Declaration of Helsinki.

### Study design

Our study design was outlined as follows:

1. Collect objective dry eye parameters in DED patients, both with and without primary Sjogren's syndrome.
2. Grade the results of tear MMP-9 in InflammaDry using a 5-scale grading system and a dichotomous grading system (D1 to D4).
3. Analyze the differences in collected objective dry eye parameters (1) based on the tear MMP-9 grades (2).
4. Identify cut-off values of objective dry eye parameters that reflect the positivity of tear MMP-9 in InflammaDry.

### Subjects

We included the medical records of 237 patients (464 eyes) who visited the Department of Ophthalmology at Chung-Ang University Hospital between March 2019 and January 2020 and were diagnosed with DED based on the TFOS DEWS II diagnostic criteria^22^. PSS was diagnosed according to the 2016 American College of Rheumatology (ACR)/European League Against Rheumatism (EULAR) classification criteria^23^. The subjects were divided into two groups: non-SS DED group and SS DED group.

### DED parameters

As part of our assessment of DED parameters, we evaluated corneal sensitivity scores using the Cochet-Bonnet esthesiometer, Tosm, tear MMP-9 levels, tear secretion with Schirmer I without anesthesia, tear BUT, SICCA OSS, corneal erosion scores according to the National Eye Institute/Industry (NEI) grading scale, meibomian gland (MG) expressibility, and meibum quality of the secreted meibum. Additionally, we used the OSDI questionnaire to assess the subjective ocular symptoms of dry eye disease and its effect on vision-related function^24^.

Corneal sensitivity was assessed using a Cochet-Bonnet esthesiometer (Luneau Ophthalmology, Chartres Cedex, France). Starting at the longest length of 6 cm, the test involved gradually decreasing the length of the monofilament that touched the center of the cornea by 0.5 cm until the patient felt discomfort. Tosm was measured using an I-PEN (I-MED Pharma Inc., Montreal, QC, Canada) that was soaked with tear at the lower conjunctival fornix and then assembled into an analyzer that displayed test results in digits. Tear secretion was evaluated using the Schirmer I test, which involved placing a Schirmer standard strip (Eagle Vision, Memphis, TN, USA) on the outer 1/3 point of the lower conjunctival fornix and allowing tear fluid to be absorbed for 5 min, without using any analgesic eyedrops. The tear BUT was measured more than 15 min after the Schirmer I test, following established procedures^25^. A drop of normal saline was placed on a strip paper coated with fluorescein dye (Haag-Streit International, Koniz, Switzerland), which was then shaken off. The strip was gently applied to the lower lid margin to stain the tear film, and the time when the first tear film break was observed under a cobalt blue filter after the last blink was considered the BUT. The measurement was repeated three times using a stopwatch, and the average value was used.

The ocular staining score was evaluated by examining each eye with a slit-lamp under a yellow filter after fluorescein instillation^26^. The SICCA score^27^ and NEI score^28^ were obtained using established standards. Evaluation for MGD was performed in two ways: assessing MG expressibility of five glands in the central upper lid and the quality of secreted meibum. The MG expressibility of meibum from five glands was graded from 0 to 3, with 0 indicating all glands expressible, 1 indicating 3–4 glands expressible, 2 indicating 1–2 glands expressible, and 3 indicating no glands expressible. The quality of meibum was also graded from 0 to 3, with each score corresponding to clear, cloudy, cloudy particulate fluid, and toothpaste-like consistency according to previously established benchmarks^29^.

All DED clinical parameters were measured and evaluated consistently by a single experienced researcher (K.W.K).

### InflammaDry assay for tear MMP-9 and its grading systems

The tear MMP-9 test was performed using InflammaDry, following the instructions provided in the product documentation^10^. A sterile sample collector was used to dab multiple areas along the lower palpebral conjunctiva to collect tear fluid, which was then assembled into the immunoassay test cassette. After 20 s of activation in buffer solution, the intensity of the red line in a readout window was verified.

**Figure 2:**
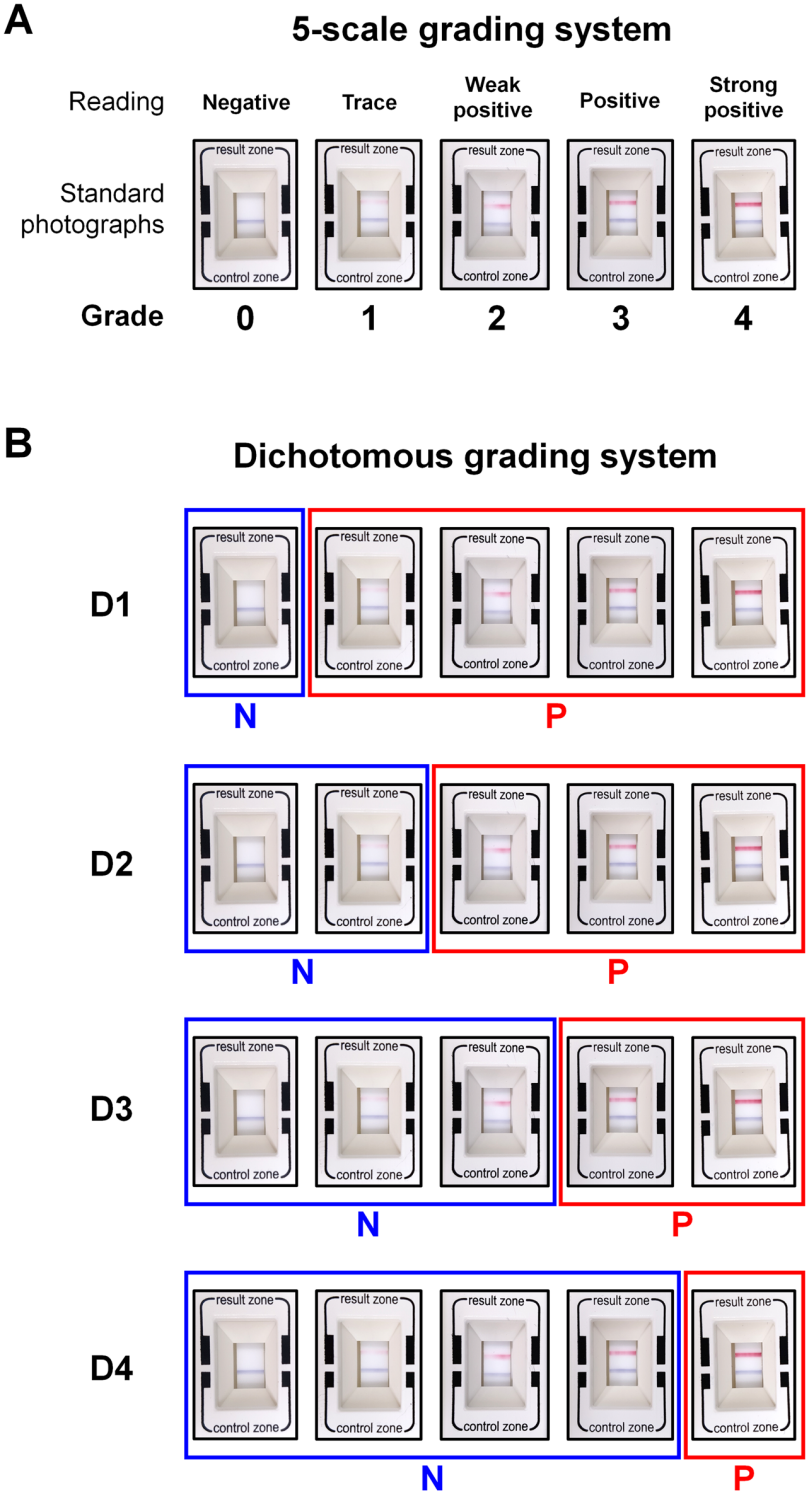
Standard photographs for tear matrix metalloproteinase (MMP)-9 expression, graded using a 5-scale grading and a dichotomous grading system (D1 to D4). (**A**) The 5-point scale ranges from 0 to 4 and is represented by the color density of the red line in the readout window of the point-of-care MMP-9 immunoassay. (**B**) The dichotomous grading system defines positive results as grades 1, 2, 3, and 4 of the 5-point scale (D1, D2, D3, and D4, respectively).

To determine the diagnostic value of the MMP-9 assay, we analyzed it using both a 5-scale grading method and a dichotomous grading method. The 5-scale grading system involved grading the depth of the red readout band of the MMP-9 on-site inspection, with grade 0 indicating a negative result, grade 1 indicating a trace result, grade 2 indicating a weak positive result, grade 3 indicating a positive result, and grade 4 indicating a strong positive result, based on previously established standard photographs (Fig. 2A)^14^. To eliminate possible interobserver variation, the 5-scale grading was performed by a single experienced researcher (K.W.K).

The dichotomous grading systems included D1, D2, D3, and D4, with the cut-off grade for a positive result being grade 1, 2, 3, and 4 by the 5-scale grading system, respectively (Fig. 2B).

### Statistical analysis

The data are presented as mean ± standard deviation. Statistical analysis was performed using GraphPad Prism v.8.1.2 (GraphPad Software, La Jolla, CA, USA). To compare variables between groups, either parametric Student’s *t*-test or non-parametric Mann–Whitney *U* test and *Chi*-square test were used. Pearson’s correlation test was used to analyze the correlation of 5-scale grades with age and ocular surface indices. Receiver operating characteristic (ROC) curve analysis was applied, and the area under the ROC curve (AUC) was calculated. A p-value less than 0.05 was considered statistically significant.

## Data Availability

The datasets generated and analyzed in the current study are available from the corresponding author on reasonable request.
